# Interpreting artificial intelligence models: a systematic review on the application of LIME and SHAP in Alzheimer’s disease detection

**DOI:** 10.1186/s40708-024-00222-1

**Published:** 2024-04-05

**Authors:** Viswan Vimbi, Noushath Shaffi, Mufti Mahmud

**Affiliations:** 1 College of Computing and Information Sciences, University of Technology and Applied Sciences, OM 311 Sohar, Sultanate of Oman; 2 Department of Computer Science, Nottingham Trent University, Nottingham, NG11 8NS UK; 3 Medical Technologies Innovation Facility, Nottingham Trent University, Nottingham, NG11 8NS UK; 4 Computing and Informatics Research Centre, Nottingham Trent University, Nottingham, NG11 8NS UK

**Keywords:** Explainable artificial intelligence, LIME, SHAP, Model agnostic, Model specific, Post-hoc anti-hoc

## Abstract

Explainable artificial intelligence (XAI) has gained much interest in recent years for its ability to explain the complex decision-making process of machine learning (ML) and deep learning (DL) models. The Local Interpretable Model-agnostic Explanations (LIME) and Shaply Additive exPlanation (SHAP) frameworks have grown as popular interpretive tools for ML and DL models. This article provides a systematic review of the application of LIME and SHAP in interpreting the detection of Alzheimer’s disease (AD). Adhering to PRISMA and Kitchenham’s guidelines, we identified 23 relevant articles and investigated these frameworks’ prospective capabilities, benefits, and challenges in depth. The results emphasise XAI’s crucial role in strengthening the trustworthiness of AI-based AD predictions. This review aims to provide fundamental capabilities of LIME and SHAP XAI frameworks in enhancing fidelity within clinical decision support systems for AD prognosis.

## Introduction

Alzheimer’s Disease (AD) is a neurodegenerative disorder characterised by the progressive deterioration of brain cells’ protein components resulting in the deposition of *plaques* and *tangles* [1]. The presence of these anomalous proteins impairs the communication between these components, resulting in a significant decline in cognitive function. Mild Cognitive Impairment (MCI) is a transitional stage from Cognitively Normal (CN) to dementia, with a 10% chance of progressing to AD [2, 3]. According to the most recent World Alzheimer’s Report, 55 million people worldwide suffer from AD, making it the seventh leading cause of death [4].

**Fig. 1:**
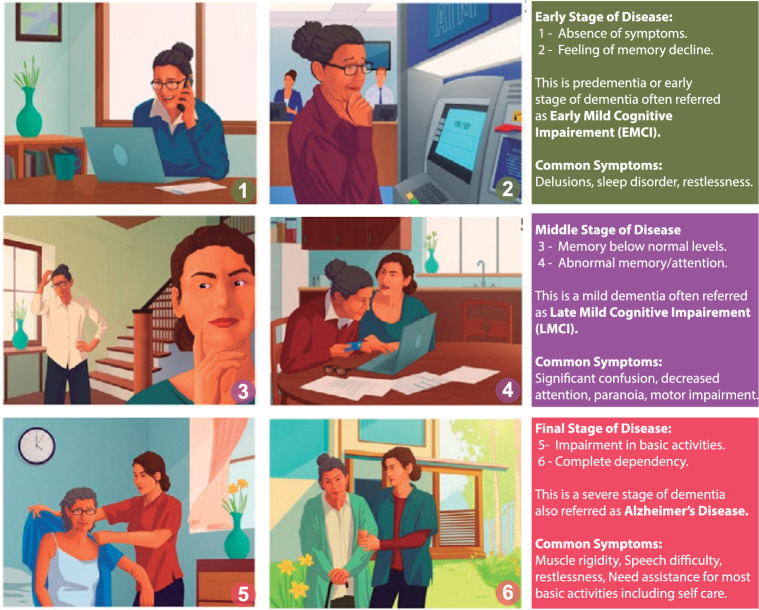
Stages of Dementia: 1–2 corresponds to early mild cognitive impairment or EMCI, 3–4 represents late mild cognitive impairment or LMCI and 5–6 depicts the AD phenomenon

Figure 1 shows different stages of dementia, which often fall into three major categories: (i) Early Mild Cognitive Impairment (EMCI), (ii) Late Mild Cognitive Impairment (LMCI), and (iii) Severe stage of cognitive impairment, which is when the patient is diagnosed to suffer from AD [4]. There are no apparent disease symptoms during the EMCI stage (frames 1 and 2), but a perceptible memory decline is observed [5]. The LMCI stage (frames 3 and 4) is marked by below-average memory and moderate dementia that has minimal effect on daily activities [6]. In this stage, the diseased cannot manage their daily affairs (such as coping with their profession) [5]. AD’s terminal stage (frames 5 and 6) is characterised by severe functional impairment that interferes with essential daily activities and necessitates frequent assistance [4, 7]. At this phase, the AD patient relies entirely on their caregiver, which causes significant physical and mental strain on the patient and their caretaking family members. MCI is a transitional stage from CN to dementia, with a 10% chance of progressing to AD. Hence, early prediction of the MCI can provide an opportunity for early intervention to prevent or delay the onset of AD.

The AD diagnosis typically takes a considerable amount of time. However, diagnostic technologies such as magnetic resonance imaging (MRI), computed tomography (CT), and positron emission tomography (PET) scans have emerged as efficient methods for collecting AD biomarkers [7]. When these biomarker data are used in conjunction with artificial intelligence (AI), it can aid in early disease prediction. In recent years AI, in particular machine learning (ML) and deep learning (DL), have attracted many researchers to contribute in diverse fields and challenging research assignments such as: anomaly detection [8–10], signal analysis [11–23], neurodevelopmental disorder assessment and classification focusing on autism [24–32], neurological disorder detection and management [33–39], supporting the detection and management of the COVID-19 pandemic [40–47], elderly monitoring and care [48], cyber security and trust management [49–54], ultrasound image [55], various disease detection and management [56–63], smart healthcare service delivery [64–66], text and social media mining [67–69], understanding student engagement [70, 71], etc. ML and DL models have also been used extensively in AD prediction due to their ability to analyse large amounts of data and identify patterns that may not be immediately apparent to human experts [7, 37, 72–77]. ML and DL models can identify patterns and signals that may indicate the early stages of a disease, allowing for early detection and treatment. DL models are even more popular, and the results obtained for AD prediction by DL models are unparalleled to this date [6, 78–80].

While ML and DL models have shown great promise in AD prediction, their black-box nature remains a significant hurdle to their adoption in real-world scenarios [81]. The lack of interpretability and transparency can lead to reluctance by medical professionals to use these models in real-world scenarios [82]. For instance, if a model predicts that a patient is at high risk for AD, the physician needs to know the reasons behind the prediction to make informed decisions about treatment and care.

**Fig. 2:**
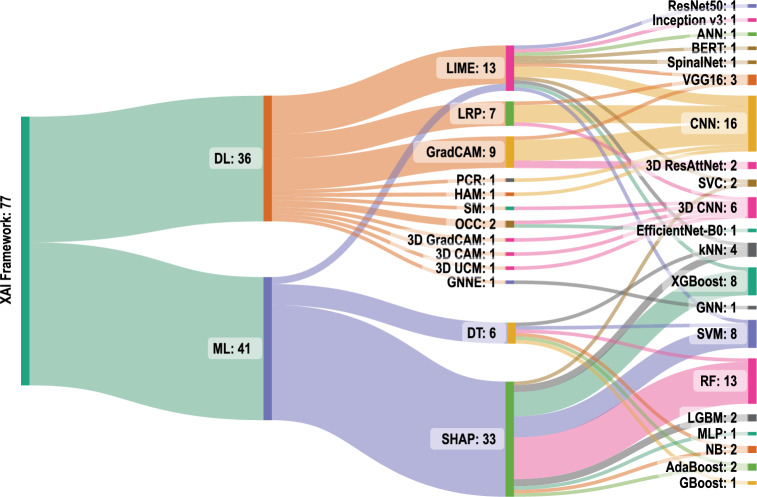
Sanky Diagram of various XAI frameworks used in AD prediction since 2018–2023 September. Reproduced with permission from [83]

Hence, Explainable Artificial Intelligence (XAI) is gaining importance in recent years which refers to techniques and methods used to make AI models more transparent and interpretable [30, 81, 81, 83]. Some examples of XAI techniques include saliency maps and feature importance analysis. Of many different XAI techniques, LIME and SHAP remain popular for explaining ML and DL models in AD prediction [83]. Based on the data presented in Fig. 2, it can be inferred that LIME and SHAP tools have been the most popular XAI frameworks for AD prediction and interpretation, with nearly 70% of the studies utilising them [79]. Hence, a comprehensive review article covering the broad scope of these techniques is imperative.

This review article covers various aspects, such as the theoretical foundations and implementation of these techniques, their applications in AD classification, and potential benefits associated with their use. Furthermore, the review article also explores these techniques’ limitations and discusses possible future research directions.

**Table 1:** Comparing LIME and SHAP frameworks

Criteria	LIME	SHAP
Explanation scope	Focus on local interpretability	Offers global and local insights
Implementation and applicability	Local, global, model-agnostic	Local, global, model-agnostic
	Post-hoc	Post-hoc
Explanation type	Textual, visual	Numeric, visual
Github link	https://github.com/marcotcr/lime	https://github.com/slundberg/shap

This resource would be an excellent reference point for researchers and professionals who seek to examine deeper into the XAI frameworks and develop accurate and interpretable models for AD diagnosis and classification.

This study makes three notable contributions:

1. *Methodological Excellence:* The research employs a systematic review methodology aligned with the guidelines proposed by Kitchenham [84] and PRISMA [85], ensuring a rigorous and comprehensive analysis.
2. *In-Depth Exploration:* The formulation of research questions (RQ) addresses the holistic landscape of LIME and SHAP XAI frameworks for AD classification. The study conducts a thorough survey of these methods over the last decade, critically analysing their findings, results, capabilities, and limitations.
3. *Practical Guidance:* The study goes beyond theoretical analysis by providing Python-based code walkthroughs for implementing LIME and SHAP frameworks. This practical guidance is especially beneficial for newcomers entering the field, enhancing accessibility and application of the presented frameworks.

The rest of the paper is structured as follows: Sect. 2 provides a brief overview of LIME and SHAP XAI frameworks. The search strategy is explained in Sect. 3. Section 4 presents the findings of this systematic review and Sect. 5 draws the concluding remarks.

## Overview of SHAP and LIME

The predictions of machine learning algorithms, particularly for medical diagnosis, can be disastrous if acted upon with blind faith. The models are evaluated based on accuracy metrics. Besides using accuracy metrics, inspecting each prediction and interpreting significant instances that lead to the decision is necessary [83]. Such explanations for instances of individual predictions can lead to trusting the prediction [83]. Multiple such predictions and explanations can help trust the model. LIME and SHAP are popular model interpretability frameworks featuring various approaches. While LIME focuses on local interpretability, SHAP offers global and local insights with dual interpretability.

Table 1 provides key distinctions between the LIME and SHAP XAI frameworks. For an in-depth understanding of XAI-specific terminologies, readers should refer to recent review articles on XAI [81, 83]. This section furnishes a brief overview of these frameworks.

**Fig. 3:**
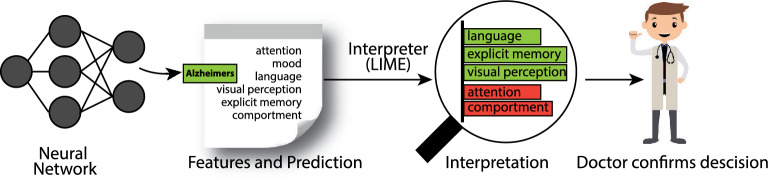
A model predicting a patient with AD and LIME highlights the symptoms that led to the prediction

### Local interpretable model-agnostic explanations (LIME)

LIME is an algorithm that, by locally approximating any classifier or regressor with an interpretable model, can accurately explain the predictions of any classifier or regressor [86]. Interpretable representation and local fidelity are two essential characteristics of LIME. Interpretability provides a qualitative understanding between the input variables and the responses. At the same time, local fidelity corresponds to the trustworthiness or faithfulness of the model’s performance within the vicinity of the predicted instance. The term model-agnostic implies that the explainer algorithm can explain any model by treating the original model as a black box model [83]. LIME can interpret image classifications, explain text-based models, and provide explanations for tabular datasets. These explanations can be presented in different forms, including textual (see Fig. 9), numeric, or visual formats. As shown in Fig. 3, an interpretable model is easily understood by humans irrespective of the model’s basic feature set. For instance, in image classification for AD, the classifier may represent the image as a tensor with three colour channels per pixel. Then, an interpretable representation can be a binary vector indicating the presence or absence of a contiguous patch of pixels that can explain the prediction.

**Algorithm 1:**
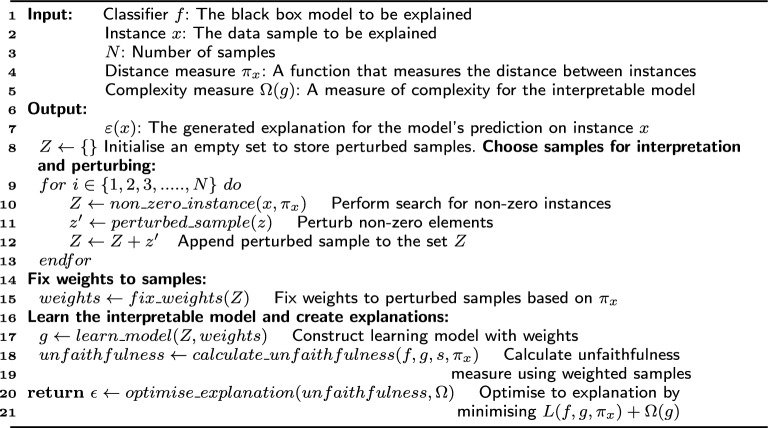
Explanations using LIME

The listing in Algorithm 1 presents the step-by-step approach to realising LIME explanations. Suppose *G* is a class of interpretable models and $g\in G$ is a model that can be readily presented with visual or textual artefacts. In that case, the domain of g is $\{0, 1\}^d$, indicating the presence or absence of interpretable components. However, not every $g\in G$ may be interpretable, so let $\Omega (g)$ be a measure of complexity. For a model $f: {\mathbb {R}}^d \rightarrow {\mathbb {R}}$ that needs interpretability, *f*(*x*) denotes the probability of *x* belonging to a specific class. To define the locality around *x*, let $\pi _x(z)$ be a measure of the distance between an instance *z* and *x*. Finally, let ${\mathcal {L}}(f, g, \pi _x)$ denote a measure of the unfaithfulness of *g* in getting an explanation for *f* within the locality defined by $\pi _x$. These parameters are used as input as shown in Algorithm 1 to obtain an explanation for LIME represented by Eq. 1:

1 $$\begin{aligned} \epsilon (x) = {\text {argmin}}_{g \in G} {\mathcal {L}}(f, g, \pi _x)+\Omega (g) \end{aligned}$$

where ${\mathcal {L}}(f, g, \pi _x)$ and $\Omega (g)$ must be minimised to ensure interpretability and local fidelity.

**Fig. 4:**
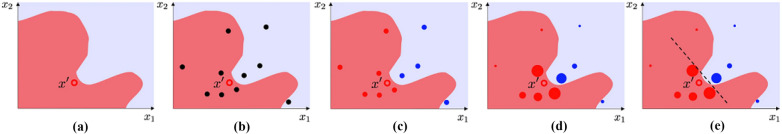
**a** Binary classification task from two features. **b** Randomly perturbed data sample. **c** Perturbed samples labelled using a black box model. **d** LIME weights samples based on proximity. **e** LIME learns linear model (best when visualised in color)

An empty set *Z* is initialised (step 8) to store the non-zero instances chosen from a linear model, for example, drawn by minimising Delta ${\mathcal {L}}(f, g, \pi _x)$ weighted by $\pi _x$ around $x'$ (see Fig. 4a). From steps 10–14, it is seen that the *N* data samples around $x'$ are randomly perturbed. The perturbed samples can be represented as $z'\in \{0, 1\}$ d’ and contain some non-zero elements of $x'$. The original representation of the sample can be reformulated as $z \in {\mathbb {R}}^d$. In classification, *f*(*z*) is the probability or binary indicator that *z* belongs to a particular class. These perturbed samples are append to the set *Z* and again fed to the black box model, and *f*(*z*) is used to obtain the classification labels (see Fig. 4b, c).

The next step is to fix weights to the chosen samples (Refer Algorithm 1 step 16). The primary intuition behind LIME is building a good local approximation using $\pi _x$ where samples with higher weight lie near $x'$ and others (with lower weight) far from $x'$. Therefore, to learn the interpretable model, LIME again fixes weights to the perturbed samples according to their proximity to $x'$. Samples close to $x'$ are given a more significant weight, and samples far from $x'$ are given low weights (see Fig. 4d). The model with perturbed data samples *Z* s used to construct a learning model by adding weights $g(z') = wg \times z'$ and the new function of unfaithfulness ${\mathcal {L}}$ found as in Eq. 2:

2 $$\begin{aligned} {\mathcal {L}}(f, g, \pi _x) = \sum _{z, z' \in Z} \pi _x(z) (f(z) - g(z'))^2 \end{aligned}$$

where the weight $\pi _x (z) = e^{\frac{-D(x,z)^2}{\sigma ^2}}$ defined on some distance function *D* based on the type of resultant artefacts (textual or visual) with width $\sigma$.

Given this dataset *Z* of perturbed and weighted samples with associated labels, Eq. 1 is further optimised to get an explanation $\epsilon (x)$ (see Algorithm 1 step 21). Considering a default linear model for LIME with sparse features, learning from the weighted samples provides adequate explanations for the prediction that $x'$ is intrinsically interpretable. The model’s linear weights can be seen as feature scores indicating its importance in prediction (see Fig. 4e).

### SHapley additive explanations (SHAP)

SHAP is an XAI technique based on a mathematical method that assigns a weight called the Shapley value, to each feature of a trained model [87]. The weight assigned to each feature measures its contribution to the prediction and is based on game theory concepts. SHAP is a model-agnostic explainer that is an interpretable model by itself. It can predict the original black box model for a specific data instance by determining the essential features and their influence on the model prediction.

**Algorithm 2:**
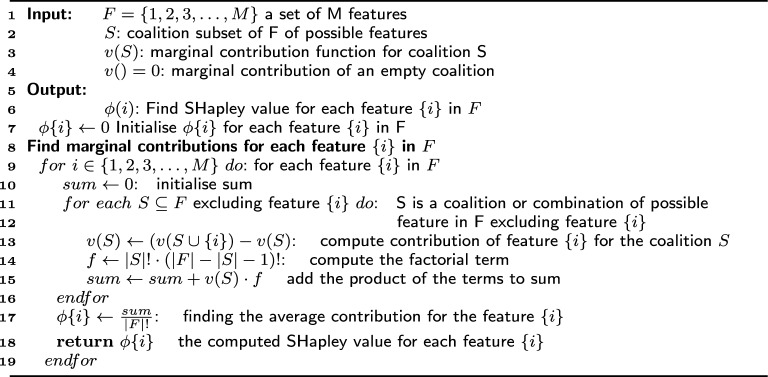
Explanation using SHapley values ($\phi$)

In this section, we explain SHAP as a simple linear regression machine learning model that predicts the absence or presence of a disease. We assume *F* as a set of *M* features $\{1, 2, 3,\cdots ,M\}$, a coalition or combination of possible features, *S* as a subset of *F* $(S\subseteq F)$, and $\phi$ as an empty set (coalition with no features). Then based on cardinality $2^M$ is the possible number of coalitions. We also assume a function *v* that maps each coalition to a real number called the marginal contribution of the coalition (see Algorithm 2). Then, the marginal contribution is *v*(*S*) for each coalition *S*, and for an empty coalition, it is given by Eq. 3:

3 $$\begin{aligned} v(\phi ) = 0 \end{aligned}$$

For each permutation *P*, the first step is to calculate the marginal contribution of the coalition of features *S*, which were added before a feature $\{i\}$ (Refer steps 11–16 from Algorithm 2). Subsequently, the coalition’s contribution formed by adding the feature $\{i\}$ to *S*, which is the coalition $S \cup \{i\}$, is found. In Eq. 4, the contribution of the feature $\{i\}$ is represented as $\phi (i)$:

4 $$\begin{aligned} \phi (i) = \frac{1}{|F|!} \sum _{P} (v(S\cup \{i\}) - v(S)) \end{aligned}$$

where |*F*| is the number of features of set *F*, |*F*|! is the total number of permutations of the coalition set *F* (consisting of all features) and $v(S\cup \{i\})--v(S)$ is the contribution of the feature $\{i\}$ to the total contribution of each permutation. In Eq. 4, the sum of the contributions is divided by |*F*|! to find the average contribution for the feature $\{i\}$. Therefore, the total contribution of the feature $\{i\}$ to the total contribution of all permutations for one possible coalition *S* in *F* is given by Eq. 5:

5 $$\begin{aligned} |S|! \cdot (|F| - |S| - 1)! \cdot (v(S\cup \{i\})-v(S)) \end{aligned}$$

The process can be repeated for other coalitions of $F-\{i\}$ to obtain the sum of the contributions of the feature $\{i\}$ in all the permutations of *F* as in Eq. 6 (see steps 11 to 16 from Algorithm 2):

6 $$\begin{aligned} \sum _{S \subseteq F-\{i\}} |S|! \cdot (|F| - |S| - 1)! \cdot (v(S\cup \{i\})-v(S)) \end{aligned}$$

Finally, considering the |*F*|! permutations for *F*, the average contribution of the feature $\{i\}$ to the total contribution of all the permutations of *F* is given by Eq. 7:

7 $$\begin{aligned} \phi (i) = \sum _{S \subseteq F-\{i\}} \frac{|S|! \cdot (|F| - |S| - 1)!}{|F|!} (v(S\cup \{i\})-v(S)) \end{aligned}$$

where $\phi (i)$ is the Shapley value for one feature $\{i\}$ and is the mathematically computed marginal contribution of the feature $\{i\}$ to the total contributions of all the features in *F*. The process can be repeated to compute the Shapley values for every other feature $\{i\}$ and represent that feature’s contribution to the model output for a specific prediction (see steps 17 and 18 from Algorithm 2).

For example, considering input features for AD, like age, gender, education level, cognitive test scores, and brain image data and aggregating the SHAP values for the entire dataset, we may find that the cognitive test scores have the highest negative SHAP value, indicating that they are strongly associated with a lower probability of AD. On the other hand, the age, education level, and brain imaging data can have positive SHAP values, indicating that they are associated with a higher probability of AD. Further, the SHAP values can be visualised using various plots, such as a summary plot (see Fig. 10) that shows the global importance of each feature or a force plot (see Fig. 11) that shows the contribution of each feature to a model prediction.

## Research questions and search strategy

**Fig. 5:**
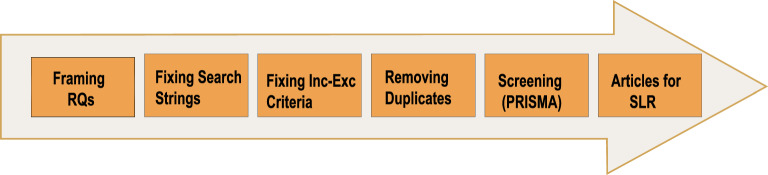
Process of relevant article identification

We followed PRISMA [85] and Kitchenham [84] guidelines to identify relevant papers for this review. The overall process is shown in Fig. 5.

The first process is framing clear and well-defined Research Questions (RQ). This ensures that the review is focused, helps to guide the search for relevant studies, and aids in data extraction and synthesis. The RQs used in this study are shown in Table 2. Next, appropriate search strings are finalised by developing a list of relevant keywords and synonyms. The search strings shown in Table 3 are finalised after several permutation combinations of identified keywords. The search for relevant articles was carried out on five databases.

**Table 2:** Research Questions

RQ	Research questions	Motivation
RQ1	What AI systems are available for AD research that incorporate LIME and SHAP?	Understanding black-box models employed in AD detection that utilise LIME and SHAP for improved clinical fidelity
RQ2	What are the different input modalities used by LIME and SHAP for AD detection?	Understanding comprehensively supported input modalities for these XAI frameworks
RQ3	What are the benefits of using LIME/SHAP for AD detection?	Exploring practicality of employing XAI tools to elucidate AD predictions and their implications within the medical community
RQ4	What are the limitations and challenges, and future prospects of LIME and SHAP in AD detection?	To comprehend the fundamental capabilities and limitations, as well as to identify research gaps that prompt further research

**Table 3:** Inclusion–exclusion criteria, search strings, and scientific repositories used in data synthesis

Inclusion criteria	Exclusion criteria	Search string	Database
Studies related to AD diagnosis using AI techniques	Pilot papers, Editorials, proceedings, magazines	“Alzheimer’s” explainable AI, “Alzheimer’s” interpretable AI	IEEE Xplore (www.ieee.org), ScienceDirect (www.sciencedirect.com)
Studies related to Explainable AI for AD prediction	Articles not related to AI based AD and AD disease diagnosis	“Alzheimer” explainable ML, “Alzheimer” interpretable ML	Springer (www.springer.com), ACM (www.acm.org)
Studies related to performance results of ML/DL models for AD	Article on AD but not on detecting it (e.g., supportive care)	“Alzheimer” explainable DL, “Alzheimer” interpretable DL	PubMed (https://pubmed.ncbi.nlm.nih.gov)
Studies related to AD Explainability using LIME and SHAP		“Alzheimer” post hoc explainable AI, “Alzheimer” XAI	

**Fig. 6:**
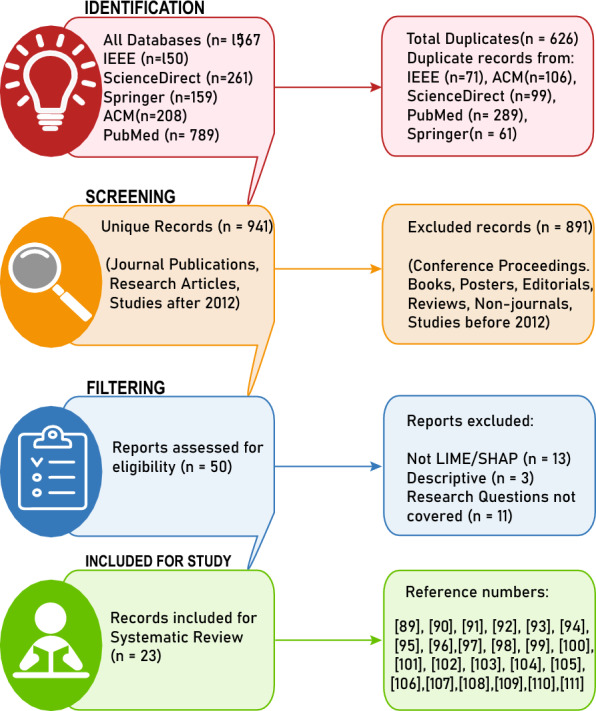
Filtering of Alzheimer’s Disease Studies with LIME and SHAP: The PRISMA Approach

Initial search until September 2023 in these databases yielded 1567 research articles (208 from ACM, 150 from IEEE, 159 from Springer, 789 from PubMed, 261 from ScienceDirect). These records were screened for duplicates, which resulted in 941 unique records. Next, the identified articles are screened using titles and abstract of publication with the help of inclusion–exclusion criteria as shown in Table 3. This effectively reduced the number of relevant articles to 50 records that exclusively dealt with XAI-based AD classification. However, this included research articles dealing with other XAI frameworks such as Gradient Class Activation Mapping (GradCAM), Layerwise Relevance Propagation (LRP), Salience Map, etc. So, a final screening included only studies using LIME and SHAP frameworks in the model interpretability, which effectively had 23 research articles. Figure 6 shows a proper understanding of the steps taken in the process.

## Data synthesis

In this section, we present our findings by extensively reviewing the 23 articles through the RQs shown in Table 2.

### LIME and SHAP XAI frameworks for AD detection

This subsection addresses the RQ1: What AI systems are available for AD research that incorporate LIME and SHAP?

**Fig. 7:**
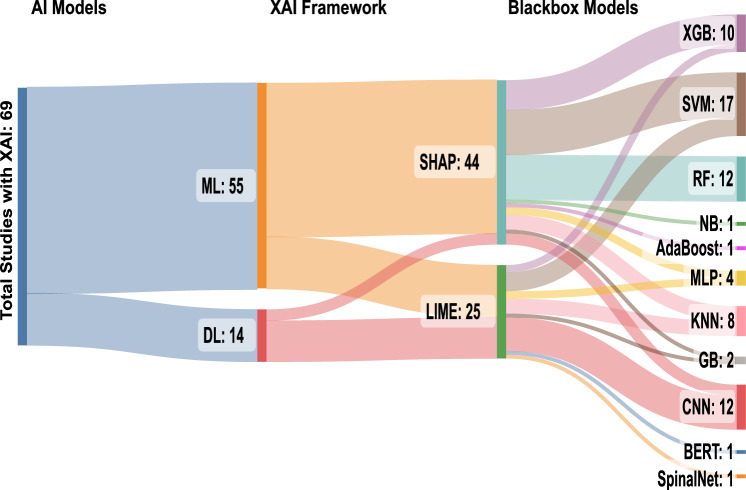
Sanky diagram of LIME and SHAP frameworks used in this review

Since 1970 there has been immense attention on AI in disease diagnosis and treatment [88] and has achieved important progress in research over the years. The concept of eXplainable AI (XAI) has recently been introduced into AI-based AD prediction which is a suite of machine learning techniques that produce models due to a growing demand for transparency and explainability in healthcare and medical practice. The XAI techniques make it possible for people to comprehend, believe in, and control the newest generations of AI models. Among the emerging techniques, two frameworks have been widely recognised as state-of-the-art in XAI and those are: the LIME framework introduced by Rebeiro et al. [86] and the SHAP values introduced by Lundberg et al. [87]. Several studies for AI-based AD detections incorporating LIME and SHAP have been identified (see Tables 4, 5 and 6, and the mapping on Fig. 7). Some of the research articles have utilised datasets that include ADNI, OASIS, and Kaggle for training AI-based AD detection models. The following subsections focus on the strengths and applications of LIME and SHAP individually. Subsequent subsection, analyses papers that integrate both techniques, exploring the combined insights they provide for enhanced interpretability in machine learning models.

**Fig. 8:**
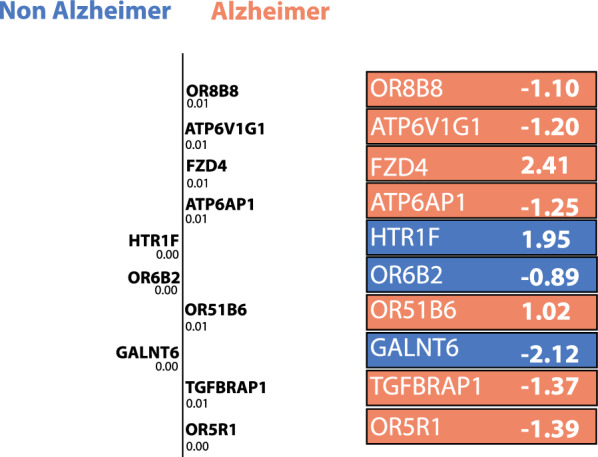
LIME Explanation (modified from [89])

**Table 4:** Studies incorporating LIME framework for explaining model predictions

References	Task	Data type	Sig. features	Classifier	Blackbox
[89]	mdDem versus moDem versus noDem versus vmDem	Image	OR8B8, ATP6V1G1	ML	SpinalNet
		Numeric	FZD4, HTR1F, OR68B2	DL	CNN, SVC
			GALNT6, ATP6AP1		XGBoost, KNN
			TGFBRAP1, ORGR1		
[90]	noDem versus vmDem	Image	Super pixel generation	DL	VGG16, CNN, ResNet50, Inception v3
[91]	MCI versus AD	Numeric	Headplot	ML	SVM, ANN
			Spectogram	DL	CNN
[92]	HC versus AD	Categoric	Text	DL	BERT, BioBERT
			Vocubulary		BioClinicalBERT
			Word		RoBERTa, ALBERT
			Linguistic		XLNet, MTL-BERT
					ConvBERT
					MTL-BERT-DE
[93]	noDem versus vmDem versus mdDem versus moDem	Image	Super pixel generation	DL	CNN

#### Studies based on LIME

This section focuses on review articles using LIME, known for its model-agnostic local interpretability, and generates explanations around instances using perturbation (see Table 4). Hamza et al. [90] experimented with neural network models for early AD detection by employing classification approaches utilising a hybrid dataset from Kaggle and OASIS. In this study, the LIME explainer is used to explore the exact region for which a specific classification occurs. The predicted result is perturbed to create featured data. A local linear model is obtained that includes partial value moderation. LIME now interprets the probable outcome of the newly generated data by assigning weights in the model to justify the prediction of AD patients whether it is in the early stage or later. Kamal et al. [89] have used images and gene expression to classify AD and also explained the results in a trustworthy way. In this study, LIME interprets how genes were predicted and which genes are particularly responsible for an AD patient. The genes identified for AD are ranked based on probability values and are separated into AD and non-AD classifications. Figure 8 shows an illustration of the LIME explanation from this study. Another article by Loukas et al. [92] has used speech recordings and associated transcripts from the ADReSS Challenge dataset to detect AD. In this article, LIME was employed to explain the BERT model that shed light on the differences in language between AD and non-AD patients. Maria et al. [91] propose a novel approach for classifying Electroencephalogram (EEG) signals to provide early AD diagnosis. The XAI method used in the study provides quantitative features that help arrive at the prediction using EEG recordings obtained from individuals with probable AD, MCI, and HC. Duamwan et al. [93] in their study discuss contemporary techniques like neural networks that often operate as black boxes, emphasising the importance of understanding the rationale behind predictions, particularly in the medical domain. This study uses a CNN-based computer vision method to find AD using the ADNI MRI dataset. It was able to classify unseen MRI scans with 94.96% accuracy. The LIME algorithm is used to make things easier to understand by giving visual proof and automatically showing parts of images that help make predictions through a segmentation algorithm. The primary objective of the study is to use LIME in this context to furnish medical professionals with specific, easily comprehensible information, facilitating efficient, consistent, and convenient diagnoses.

**Table 5:** Studies incorporating SHAP framework for explaining model predictions

References	Task	Data type	Sig. features	Classifier	Blackbox
[94]	HC versus sMCI versus	Numeric	Cognitive, PET, MRI, CSF	ML	RF
	pMCI versus AD		Genetics, Medical history		
			Other Individual modalities		
			Neuropsychological battery		
[95]	HC versus MCI versus AD	Numeric	Volumetric measurements	ML	RF
			Cognitive tests, ApoE allele		XGBoost
			Demographic features		
[96]	HC versus MCI versus AD	Numeric	Demographic, Clinical	ML	RF
			Neuropsychological		
[97]	HC versus MCI versus AD	Numeric	Clinical history, Cognitive features	ML	XGBoost
			Anatomical Metabolic features		SVM, RF
			CSF biomarkers,ApoE4		
[98]	HC versus AD	Numeric	Endoplasmic Reticulum stress related differentially expressed genes measures	ML	AdaBoost, RF
					LGBM, XGBoost
					kNN, NB, SVM
					LR
[99]	HC versus erMCI versus	Numeric	CDRSB, Age, MMSE, RAVLT	ML	XGBoost
	ltMCI versus AD	Categoric	measure, MRI middle temporal artery measure		RF
			Gender, ApoE		
			FDG, MRI whole brain		
			MRI entorhinal measur		
			MRI hippocampus measure		
[100]	aMCI versus AD	Numeric	Clinical, Demographic, ApoE genotype, Neuropsychological	ML	LR, RF
					XGBoost,SVM
[101]	HC versus MCI versus AD	Numeric	CDRSB, MMSE, EcogSPTotal	ML	Random Seeds
			RAVLT-perc-for-getting		SVM-SMOTE
			FAQ, ADAS11, MOCA		RF
			LDELTOTAL		
[102]	HC versus erMCI versus	Numeric	MRI Volumetric measures, Age	ML	DT, LGBM
	ltMCI versus AD	Categorical	Gender, Education, ApoE		RF, SVM
[103]	HC versus sMCI versus	Numeric	MRI Volumetric measures	ML	XGBoost
	pMCI versus AD		ApoE4 alleles, Cognitive results		RF, SVM
			Socio-demographic data		
[104]	HC versus AD	Numeric	Socio-demographic data medical history, Life Style measures	ML	RF
					XGBoost
[105]	HC versus ltMCI versus AD	Numeric	Amyloid beta features, glucose	ML	RF
			MRI measures		
[106]	noDem versus vmDem versus mdDem versus moDem	Image	Image patterns	DL	CNN
				ML	KNN, SVM
[107]	HC versus AD	Numeric	Image patterns	DL	3DCNN
		Image	Demographic and Cognitive biomarkers		
[108]	HC versus MCI	Numeric	Clinical information	ML	XGBoost, RF
			Neuropsychological test		AdaBoost, NB
			Data, Neuromaging-extracted biomarkers, gene data		
			APOE-$\epsilon$4		

#### Studies based on SHAP

In this review, it was found that SHAP is another XAI framework that is being used frequently rooted in cooperative game theory, offering a unified measure of feature importance (see Table 5). Shaker et al. [94] have developed and utilised a multi-layered multi-model system for an accurate and explainable AD diagnosis. The authors have used SHAP in each layer of the Random Forest (RF) architecture for a local and global explanation and provide a complementary justification by using several other explainers that include decision trees and fuzzy rule-based systems. Bloch et al. [95] state that the diverse causes of AD can lead to inconsistencies in disease patterns, protocols used for acquiring scans, and preprocessing errors of MRI scans resulting in improper ML classification. This study investigates whether selecting the most informative participants from the ADNI and Australian Imaging Biomarker and Lifestyle (AIBL) cohorts can enhance ML classification using an automatic and fair data valuation method based on XAI techniques. Angela et al. [96] present a robust framework for classification between CN, Mild Cognitive Impairment (MCI), and AD and interpret the predictions with XAI methods. The article shows how SHAP values can accurately characterise its effect on a patient’s cognitive status. Monica et al. [97] compare the performances of the best three models from ‘The Alzheimer’s disease prediction of Longitudinal evolution’ (TADPOLE) challenge concerning prediction and interpretability within a common XAI framework. SHAP values explain the decision made by the RF classifier for each sample with a vector showing feature importance for each subject at a specific visit. Based on interpretable machine learning, Lai et al. [98] investigate the endoplasmic reticulum (ER) stress-related gene function in AD patients and identify six feature-rich genes (RNF5, UBA C2, DNAJC10, RNF103, DDX3X, and NGLY1) that enable accurate prediction of AD progression. This article uses SHAP along with white-box models that include decision trees and Naive Bayes (NB) for a local and global interpretation of each feature within the ML models. The study by Bogdanovic et al. [99] used XGBoost and RF for a four-way classification of disease from HC, early MCI, late MCI and AD. The explainer SHAP is used here for a local and global interpretation of the model. Chun et al. [100] try to improve the predictive power of progression from amnestic MCI to AD using an interpretable ML algorithm. This study uses several classifiers including logistic regression (LR), RF, Support Vector Machine (SVM) and XGBoost to compare the predictions. The SHAP values are expressed as summary and dependence plots for a local interpretation of individual patients and also behave as model-agnostic for a global interpretation.

Xiaoqing et al. [101] propose a reliable multi-class classification model supported by XAI methods to explain the predictions accurately. The study uses Random Seeds and Nested cross-validation SVM Synthetic Minority over Sampling (SVM-SMOTE) and RF as classifiers for a multi-way prediction. In this study, SHAP values are used for both local and global interpretation. SHAP is used by Ahmed et al. [102] and Louise et al. [103] to determine the order of informative predictors in test data. ML models and their relationships were also visualised and analysed using SHAP summary plots. SHAP force plots examined the individual forecasts of chosen individuals, and the summary plots of those models primarily displayed biologically conceivable outcomes. Sameul et al. [104], used RF and XGBoost algorithms in classifying between CN and AD. The study developed an ensemble-based ML model to predict AD and explained the prediction in local and global contexts. The study also includes feature importance analysis and ranked the dominant features influential in AD. Hammond et al. [105] use the SHAP framework to identify the biomarker that is most influential in AD detection predicted by the RF classifier. The research article tries to classify subjects into different categories like CN, MCI, or AD by using SHAP values to rank the features in each layer of RF to obtain a local interpretation. The study also aggregates the rightly ranked layers of RF and compares again for a global interpretation. In the study by Yilmaz et al. [106] authors address the designing of an explainable diagnostic machine learning model for predicting AD severity levels. Utilising two open-source MRI datasets, a Convolutional Neural Network (CNN) was developed and evaluated, achieving an impressive accuracy rate of 99.9%. This outperformance underscores the potential of deep learning in meeting diagnostic standards. To enhance transparency, the SHAP framework was employed, revealing that the model’s predictions align with well-known pathological indicators of AD, thereby providing interpretability and reinforcing its diagnostic validity. A multimodal deep-learning framework, combining a 3DCNN with a bidirectional recurrent neural network (BRNN) is introduced by Rahim et al. [107]. The 3D CNN captures intra-slice features from MRI volumes, while the BRNN identifies inter-sequence patterns indicative of AD, utilising longitudinal data over a 6-month span. The study explores the impact of fusing MRI with cross-sectional biomarkers like demographic and cognitive scores. The authors used SHAP to enhance interpretability for domain experts. Results demonstrate the framework’s robustness, achieving 96% accuracy, 99% precision, 92% recall, and a 96% AUC. The fusion of MRI with demographic features enhances stability, and the explainability module provides valuable insights, accurately identifying brain regions relevant to AD diagnoses.

Fuliang et al. [108] address the class imbalance in their study, in the context of Alzheimer’s disease diagnosis, during the transition from normal cognition to mild cognitive impairment using a machine learning approach. They have used the framework, extreme gradient boosting-Shapley additive explanations (XGBoost-SHAP), that aims to handle the imbalance among different AD progression statuses and achieve multiclassification of NC, MCI, and AD. In the study clinical, neuropsychological, and neuroimaging-derived biomarker patient data collected from ADNI database is employed for feature extraction embedded into the XGBoost algorithm. To enhance interpretability, the SHAP method is coupled with XGBoost, providing insights into the impacts of model predictions. The framework achieves high sensitivity, specificity, accuracy, and area under the receiver operating characteristic curve (AUC) on all datasets. Additionally, the study provides valuable insights for clinical decision-making based on SHAP values.

**Table 6:** Studies incorporating LIME and SHAP framework for explaining model predictions

References	Task	Data type	Sig. features	Classifier	Blackbox
[109]	HC versus AD	Numeric	Normal whole brain volume	ML	SVM, KNN, MLP
		Categoric	Years of education, Socioeconomic status, Age, MMSE, Gender		
			Intracranial volume		
			Atlas scaling factor		
[110]	HC versus mdMCI versus moMCI versus AD	Numeric	Cross sectional MRI data	ML	SVM, KNN
			Longitudinal MRI data		RF, GB
[111]	HC versus AD	Numeric	Gender, hand, age, Years of education, Socioeconomic status	ML	SVM, KNN, MLP
			Mini-mental state examination		
			Clinical dementia, Estimated total intracranial volume		
			Normalised whole-brain volume		
			Atlas scaling factor		

#### Studies based on LIME and SHAP

In this section we review articles that integrate both the techniques LIME and SHAP, uncovering insights for enhanced interpretability in machine learning models (See Table 6). Loveleen et al. [109] discuss AD prediction using tree-based models. The study employed machine learning algorithms like LR, SVM, KNN, Multilayer Perceptron, and decision trees to classify patients into demented and non-demented groups. The authors introduced an explanation-driven Human–Computer Interaction (HCI) model, achieving high accuracy across algorithms and comparing performance with state-of-the-art deep learning models. To enhance interpretability, LIME and SHAP explanation algorithms were applied to black-box deep learning models. Rashmi et al. [110] diagnoses AD with various datasets and emphasises the importance of explainability beyond diagnosis. The study utilises MRI feature data, including generic information, cross-sectional MRI data, and longitudinal MRI data. In the study, the data processing methodology involves balancing data, transferring data using a Quantile Transformer, applying PCA dimension reduction for six features, and employing a meta machine learning model. The author uses SHAP and LIME as explainable tools to elucidate the diagnostic outcome. The research achieves outstanding results, with 97.6% accuracy, 95.8% precision, 97% recall, and an F1 Score of 96.8%, as a result of employing advanced data processing techniques.

Loveleen et al. [111] advocate that medical research should go in a new, and more revolutionary direction by combining deep learning and XAI and moving toward a human–computer interface (HCI) model. The proposed study uses SHAP, LIME, and DL algorithms to create a strong and understandable HCI model. The inclusion of DL algorithms, including LR (80.87%), SVM (85.8%), k-nearest neighbour (87.24%), multilayer perceptron (91.94%), and decision tree (100%), along with LIME and SHAP, opens new avenues for exploration in the medical sciences. These findings show that using an easy-to-use computer interface in decision-making processes makes the model more accurate at making predictions. This is very important for biomedical and clinical research.

### Data modalities used in LIME and SHAP XAI frameworks

This subsection addresses the RQ2: What are the different input modalities used by LIME and SHAP for AD detection?

The popular XAI frameworks, LIME and SHAP, can be applied to a wide range of input modalities for machine learning models including numeric, categorical, image, audio and time-series data. Models that use tabular data such as medical records, financial data or customer demographics are examples of numeric data modality. Predictions in textual form like natural language text, sentiment analysis or spam detection are considered categoric in nature. The input data image constitutes medical images, facial recognition, object detection, etc. for predictions by machine learning models. LIME and SHAP also analyse audio data such as speech recognition or voice authentication and time-series data such as weather forecasting and sensor data analysis. By this RQ we categorise the reviews into subsections showing articles using image, numeric or tabular data, and categoric data modality separately along with ML techniques for prediction and subsequent AD interpretations. A few articles have used either numeric and medical images alone or along with numeric and categoric data in association with DL classifiers(see Tables 4, 5, and 6)

#### Studies that use image data for explainability

As images present unique challenges, including intricate patterns and spatial relationships, we examine in this section the essential theme of model explainability tailored to interpret complex models dealing with image data. Hamza et al. [90] collected T1 weighted MRI scans from Kaggle, aiming for a four-way classification of AD predictions. Using DL architectures like ResNet50, VGG16, and InceptionV3, they explained feature importance through LIME. The weights assigned by LIME served as explanations, justifying predictions for AD patients at various stages. Simultaneously, Duamwan et al. [93] examined the contemporary neural network techniques, emphasising the need for transparent models in medical predictions. Their study, employing a CNN-based computer vision approach on the ADNI MRI dataset, achieved a notable 94.96% accuracy in classifying MRI scans. Leveraging LIME, the research enhances interpretability by visually highlighting crucial image segments. The shared objective is to provide medical professionals with specific, easily comprehensible information, streamlining diagnoses efficiently and consistently. Yilmaz et al. [106] focus on crafting an interpretable machine-learning model to predict AD severity levels. Using two open-source MRI datasets, they developed and evaluated a Convolutional Neural Network (CNN), achieving an exceptional accuracy rate of 99.9%. This outstanding performance highlights the capability of deep learning to meet diagnostic standards. To augment transparency, the study integrates the SHAP framework, revealing that the model’s predictions align with established pathological indicators of AD. This not only enhances interpretability but also reinforces the diagnostic validity of the model.

#### Studies using numeric data for explainability

Numeric data, with its quantitative nature, plays a crucial role in decoding complex algorithms and offering valuable insights. In this section we explore studies focused on numeric data for explainability, aiming to understand how researchers harness numerical information to demystify the black box nature of machine learning models, fostering transparency and accountability in artificial intelligence.

Maria et al. [91] propose a pioneering method for early AD diagnosis by classifying Electroencephalogram (EEG) signals. The study employs an XAI method, extracting quantitative features from EEG recordings of individuals with probable AD, Mild Cognitive Impairment (MCI), and Healthy Controls (HC). Numerous studies, such as [94, 101], and [105], exclusively use datasets from the ADNI database. These studies employ numeric input data derived from diverse biological and clinical measures, including MRI volumetric readings, cognitive scores, genetic data, demographic history, and laboratory test data. Machine learning classification techniques, such as RF, SVM, LR, DT, and LGB, are utilised for classifying CN and AD. Studies in [95] and [96] compare prediction accuracy using datasets from ADNI and AIBL cohorts. These studies utilise numeric input data from biological and clinical measures to train ML models like RF and XGBoost for three-way classification (CN, MCI, and AD). The SHAP framework is consistently used for either local or global explanations of features. Similarly, [103] performs a four-way classification (HC, stable MCI, progressive MCI, and AD) using numeric input data collected from ADNI, OASIS, and AIBL cohorts. Various ML models, including XGBoost, RF, SVM, LR, and Decision tree, are employed, and SHAP is used to interpret prediction results.

Studies in [97] utilise the numeric input dataset from the TADPOLE challenge and ADNI cohorts, incorporating clinical history, cognitive and anatomical data, metabolic features, and cerebrospinal fluid biomarkers. XGBoost, RF, and SVM ML models are applied for AD classification, with SHAP providing explanations. In [98], numeric gene expression data from the Gene Expression Omnibus website is used for classifying patients between CN and AD. SHAP is employed for both local and global interpretations of predictions made by various ML classifiers. Moreover, [100] utilises clinical and neuropsychological assessments from the Samsung Medical Center, South Korea, for classifying between amnestic MCI and AD. ML models, including LR, RF, SVM, and XGBoost, are used, and SHAP is applied to explain feature importance. Fuliang et al. [108] tackle class imbalance in AD diagnosis using the XGBoost-SHAP framework. Clinical, neuropsychological, and neuroimaging-derived biomarker data from ADNI are employed for feature extraction. SHAP is coupled with XGBoost to enhance interpretability, achieving high sensitivity, specificity, accuracy, and area under the receiver operating characteristic curve (AUC) on all datasets. Rashmi et al. [110] emphasises the importance of explainability beyond diagnosis, utilising various datasets for Alzheimer’s diagnosis. The study employs MRI feature data, applies advanced data processing techniques, and uses SHAP and LIME for explanation. Additionally, Loveleen et al. [111] advocate for a revolutionary direction in medical research by combining deep learning and XAI. The study uses SHAP, LIME, and deep learning algorithms to create a Human–Computer Interface (HCI) model, achieving high accuracy in predictions.

All the referenced studies underscore the significance of numeric data in enhancing the transparency and interpretability of machine learning models, particularly in the context of AD diagnosis.

**Fig. 9:**
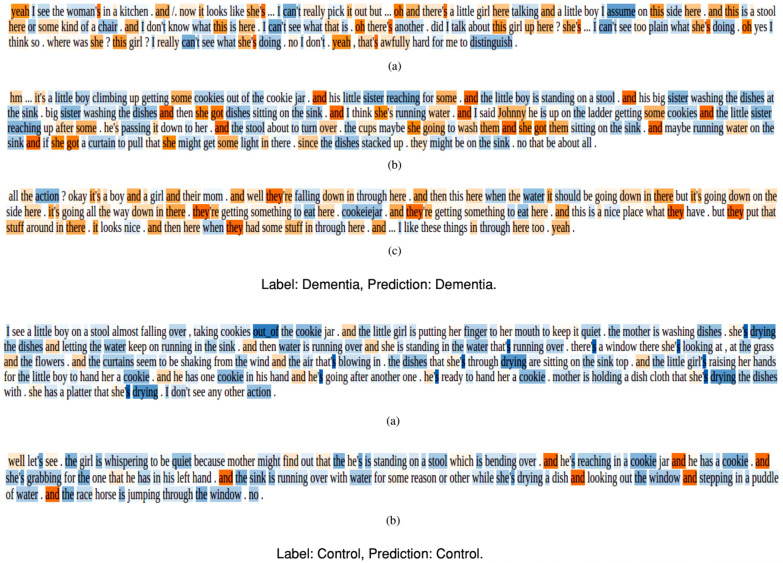
LIME Textual explanation (modified from [92])

#### Studies using categorical data 

In this review, there was only one article by Loukas et al. [92] that used a distinctive approach for AD detection. The authors used speech recordings and associated transcripts from the ADReSS Challenge dataset to detect AD. Unlike studies relying on numeric data, this investigation employs categorical data models for explainability. Specifically, Loukas et al. [92] leverage the transformer-based network BERT along with transcripts to uncover language differences between AD and non-AD patients. LIME is applied to explain the BERT model, providing insights into these linguistic distinctions. The visual representation in Fig. 9 illustrates the intensity of colours for the textual form of explanation by LIME. It suggests that AD patients exhibit a higher frequency of using personal pronouns, interjections, adverbs, verbs in the past tense, and the token “and” at the beginning of utterances. This approach offers a unique perspective by utilising categorical data and linguistic patterns for AD detection, contributing to the broader landscape of explainable AI in healthcare.

#### Studies using numeric data along with categorical or image data

Studies with an intersection of numeric, categorical, or image data are paving the way for enhanced understanding and interpretability in machine learning models. In this review, we found innovative articles that explore the combination of numeric, categorical, and image data to provide significant insights and foster transparency in machine learning outcomes.

In [99], numeric and categoric datasets from the TADPOLE challenge and ADNI cohorts, encompassing diverse clinical, anatomical, metabolic, and cerebrospinal fluid biomarker information, are employed for multi-way classification in AD. Utilising XGBoost, RF, and SVM models, the study integrates the SHAP framework for both local and global interpretations of feature importance. Despite the decision tree-based nature of ML models, SHAP’s model-agnostic attributes facilitate its extension to diverse ML models. The authors discuss potential selection bias with the Data Shapley method, emphasising more specific and less generalisable models for a particular subgroup. Figure 11 illustrates force plots depicting the impact of SHAP values on feature interaction and overall predictions at the individual level. Additionally, the study underscores SHAP as supplementary knowledge for clinicians, enhancing diagnostic conclusions over time. Analysing SHAP plots, the study identifies CDRSB as the most impactful feature, while gender and APOE4 exhibit minimal influence, challenging gender predisposition notions. The study concludes that MMSE value predominantly impacts CN subjects, with age holding the most influence on late MCI class, rendering gender insignificant. In another study, Ahmed et al. [102] utilise SHAP to ascertain the sequence of informative predictors in test data. ML models, such as DT, Light Gradient Boosting (LGB), Logistic Regression (LR), RF, and SVM, are examined using SHAP summary plots. The studies focus on CN and AD classification, employing numeric input data from biological and categoric measures obtained from the ADNI database. In the study, SHAP is applied for both local and global interpretations of feature importance. Various ML models are also explored for a 4-way classification, leveraging SHAP to establish rankings. The study further quantifies associated predictors using a proxy PCA, contributing to stable rankings.

A few studies have employed numeric and image data for AD classification and explaining thereafter. Kamal et al. [89] employed both DL and ML classifiers in a comprehensive four-way classification of AD predictions. DL classifiers, specifically SpinalNet and CNN, utilised MRI scans from Kaggle and OASIS-3, while ML classifiers, including SVM, KNN, and XGBoost, leveraged gene microarray data from the NCBI database. By combining MRI and gene expression data, the authors created a multimodal diagnostic model for AD. LIME was integrated to provide interpretability, explaining the role of genes and ranking them based on probability values in AD prediction. In a similar study, Rahim et al. [107] introduced a multimodal deep-learning framework, combining a 3D CNN with a bidirectional recurrent neural network (BRNN). This framework captured intra-slice features from MRI volumes and identified inter-sequence patterns indicative of AD, utilising longitudinal data over a 6-month span. The study explored the fusion of MRI with cross-sectional biomarkers such as demographic and cognitive scores. SHAP was employed to enhance interpretability for domain experts. Results demonstrated the framework’s robustness, achieving 96% accuracy, 99% precision, 92% recall, and a 96% AUC. The fusion of MRI with demographic features enhanced stability, and the explainability module provided valuable insights by accurately identifying brain regions relevant to AD diagnoses.

In summary, studies combining numeric, categorical, and image data for AD classification utilise diverse machine learning models. XAI methods like SHAP and LIME enhance interpretability, shedding light on influential features. This holistic approach, integrating different modalities, aims to create comprehensive and transparent AD diagnostic models, ultimately advancing our understanding of the disease and aiding clinical decision-making.

### Benefits of LIME and SHAP in AD detection

This subsection addresses the RQ3: What are the benefits of using LIME/SHAP for AD detection and in general healthcare?

Several benefits have been reported by studies in this review that use the concept of LIME and SHAP explainers in AI-based AD detection. A majority of the studies discuss the importance of adding trustworthiness in AI predictions, particularly in the medical industry. We discuss the benefits in terms of various output forms of explanations such as numeric, textual, visual and rule-based forms. There are no studies that have produced Rule-based explanations in this review. Therefore in this section, we discuss the benefits in terms of the Numeric, Textual, and Visual forms of explanations.

Kamal et al. [89] have found that LIME was useful in discovering critical genes responsible for AD. Also, the XAI method was useful in identifying the major sets of genes and their role in favouring the progress of AD disease. The authors found that the genes OR8B8 and ATP6V1G1 are found to be highly significant for AD and HTR1F and OR6B2 for non-AD patients. Hamza et al. [90] and Sidulova et al. [91] use LIME to visualise the more red areas of the brain that were identified as representative features for AD diagnosis. The colourful areas signify regions that instigate the image classification models to make the prediction. The author also finds LIME to be beneficial to comprehend low-level data. Loukas et al. [92] use the transformer-based network - BERT along with transcripts to produce differences in language between AD and non-AD patients. Figure 9 depicts the intensity of colours for the textual form of explanation by LIME, suggesting that AD patients tend to use personal pronouns, interjections, adverbs, and verbs in the past tense and the token “and” at the beginning of utterances in a high frequency.

The RF classifier is found to be used in several research along with the SHAP explainer supporting the predictions with visual explanations such as violin, force, and summary plots [94, 96, 105]. The authors in [94] claim high-performance measures for the tradeoff between accuracy and interpretability. Several credible and trustworthy visual justifications support the results.

**Fig. 10:**
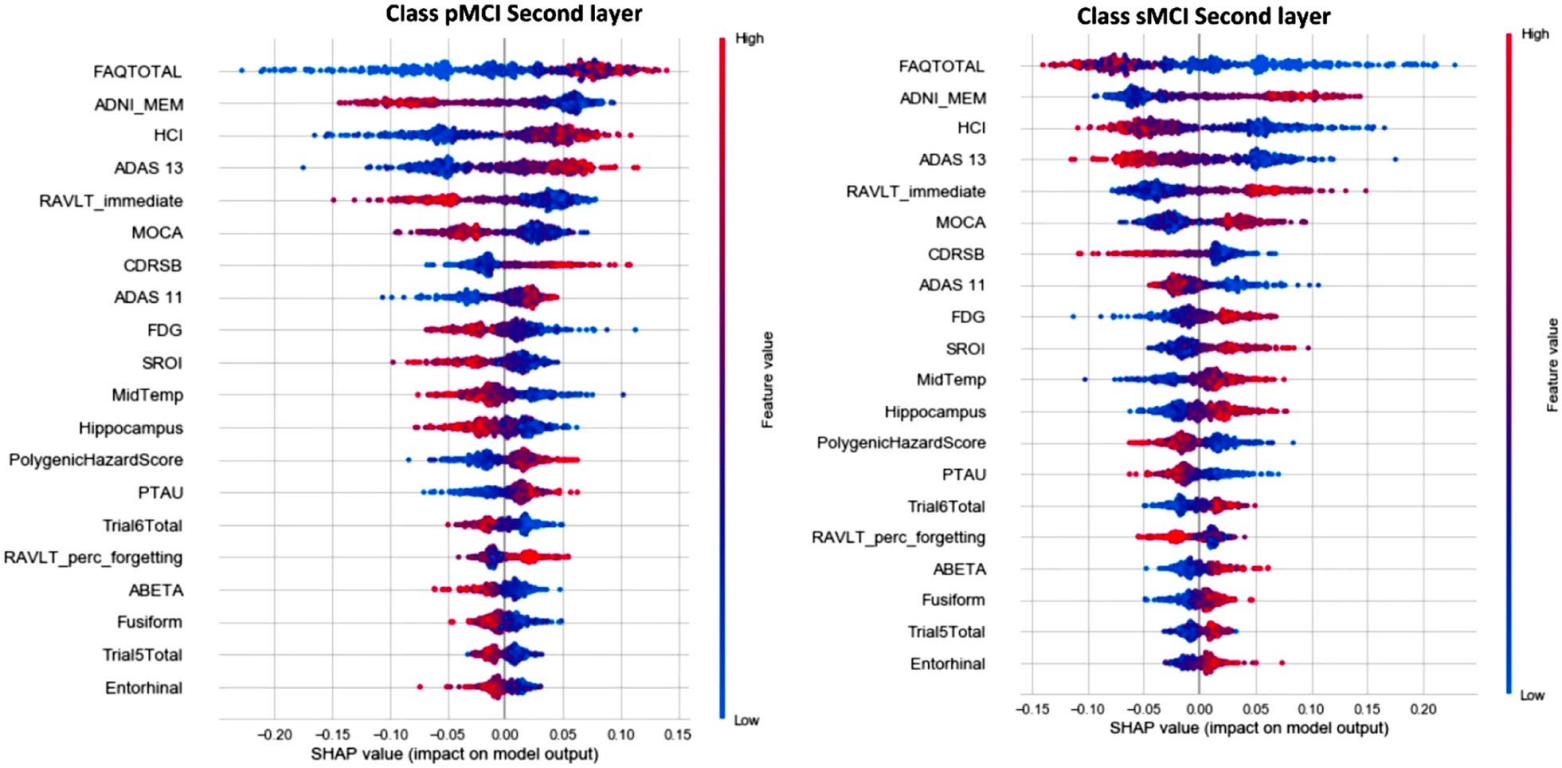
SHAP Summary Plot explanation (modified from [94])

Figure 10 shows the summary plot for the second layer for the pMCI and sMCI classes respectively. The study in [96] gives the absolute value of each SHAP score that expresses how much each feature contributes to the final prediction. The authors also show how SHAP has achieved in explaining the internals of the RF classifier trained on cognitive and clinical information. The explanations provide a possible link between diagnosis and patterns of feature relevancy. In another study [105], the authors illustrate models with high-performance measures as the models work by merging many decision trees to obtain a final global forecast. The authors replicated the analysis using SHAP and obtained a consistent feature ranking analysis. The study employed AD biomarkers that are powerful enough to predict CN, late MCI and AD and also ranked the biomarkers in order of their feature importance. The study also shows that the Amyloid beta (A), tau (T), and neurogenerative biomarkers (N) have different importance in predicting clinical dementia. The study proves the high importance of the biomarkers (A) and (T) in predicting early cognitive impairment and the glucose uptake in predicting later cognitive impairment. The authors also demonstrate a framework integrating A/T/N biomarkers using RF to classify dementia and rank biomarker features.

**Fig. 11:**
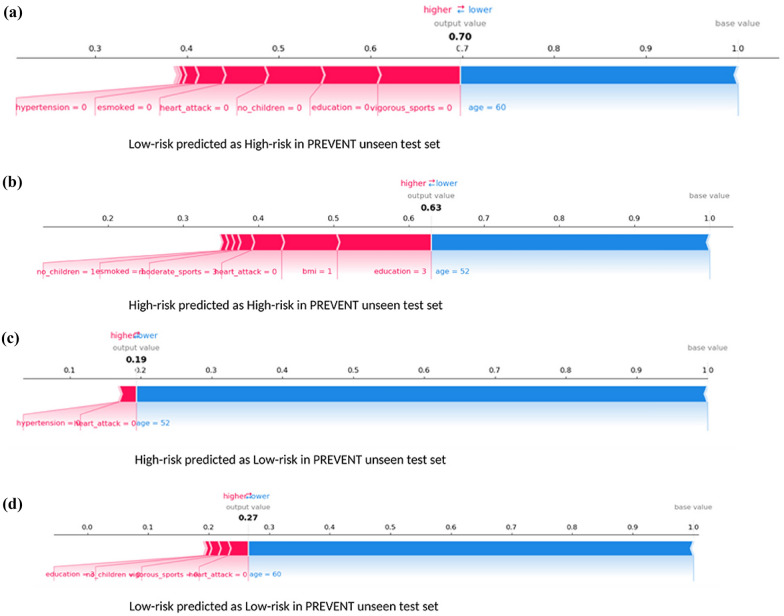
SHAP Force Plot explanation (modified from [104])

The ML models XGBoost and RF are used in [95, 99, 104] for AD classification and interpreting with SHAP. Although the ML models used in the study are decision tree-based, the SHAP model-agnostic interpreter simplified its possibility of extending the application to other ML models. The authors discuss the increased possibility of a selection bias using the Data Shapley method, leading to more specific and less generalised models and reducing the problem to a specific subgroup. Fig. 11 shows force plots with the effect of SHAP values on the interaction of features and the overall prediction at the individual level. In a different study [99], the authors demonstrate the use of SHAP as additional knowledge for clinicians and other related experts when concluding the diagnosis for a particular patient. The study claims worthy benefits regarding the model’s exactness and validity for the time difference. The study establishes, by analyzing the SHAP plot, that CDRSB leads by far the most in the impact of the model’s output. The gender and APOE4 have very low feature importance values, indicating the least influence on the prediction outcome. The authors establish that there is no gender predisposition for obtaining AD. From this outcome, it can be confirmed that the APOE gene does not act as a decisive factor in a diagnosis. The authors conclude in the study that the MMSE value impacts most on the CN subjects, and the subject’s age has the most influence on the late MCI class, leaving the gender feature insignificant.

**Fig. 12:**
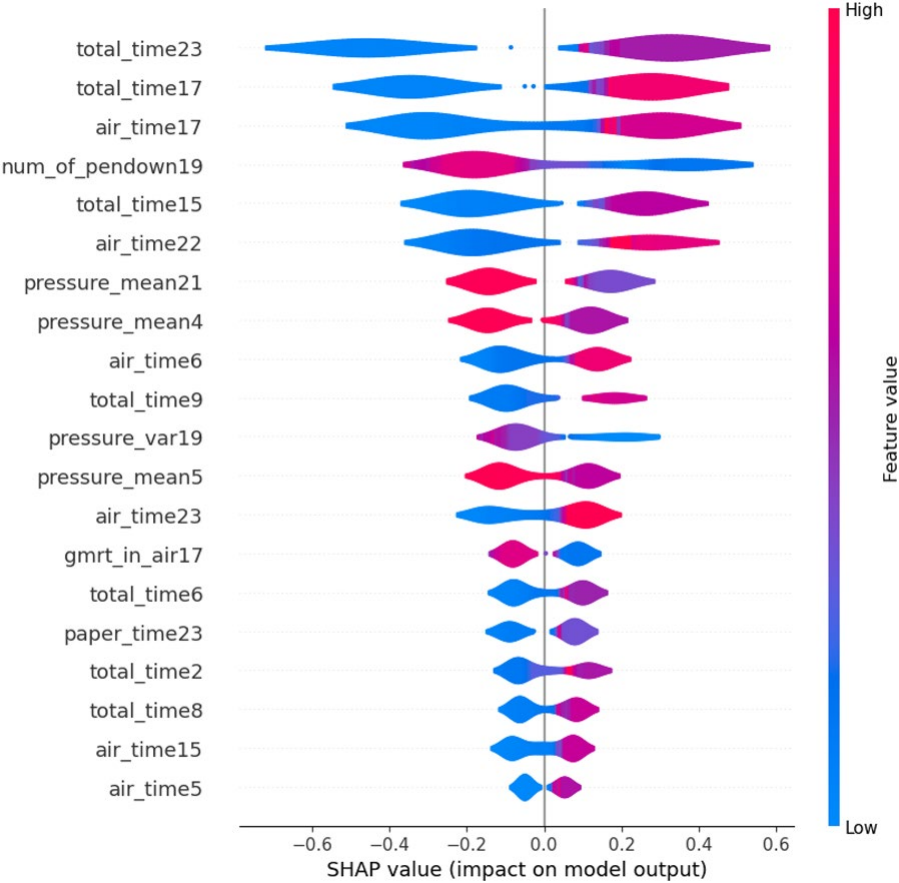
SHAP Violin Plot explanation (modified from [97])

The study by Monica et al. [97] and Min et al. [100] use the ML models LR, SVM, and RF to compare prediction performances between CN and AD. In [97], the author shows how to quantify the contribution of each feature to achieve the best accuracy and also identify features with significant importance that resulted in the prediction. The authors justified the best ML method that uses information coherent with clinical knowledge using SHAP violin plots. Fig. 12 shows the SHAP values computed from the RF classifier as described by the authors in [97]. The study [100] using LR, SVM, and RF proves noteworthy in demonstrating that the interpretable machine learning (IML) algorithm can estimate the individual risk of conversion to dementia in each MCI patient. Another major finding of the authors was that the IML, consisting of ICE and SHAP, allowed for the interpretation of variables that acted as important factors in the conversion to dementia in each patient. Altogether, both the findings in the study suggest that an algorithm using the IML technique enabled the authors to individually predict the conversion of patients with amnestic MCI to dementia.

The study by Xaioqing et al. [101] involves ML models that include Random Seeds and Nested Cross-validation, SVM-SMOTE, and RF for a three-way classification. The study uses SHAP to identify the important features among CDRSB, MMSE, EcogSPTotal, and RAVLT_perc_forgetting and distribute them according to the class. The study shows how SHAP provides a colored visual explanation for a single instance in AD class as a cumulative effect of cognitive score features based on its contribution to the class. SHAP takes explanations for each case of the test set, rotates them 90 degrees, and stacks them horizontally to visualise the test set explanations. The study proves beneficial to physicians in providing insight into why the model makes decisions. Another study by Ahmed et al. [102] uses DT, LGB, LR, RF, and SVM for 4-way classification. The authors used SHAP to derive the rankings of informative predictors in descending order. The study utilised SHAP and proxy PCA to measure predictors, which produced uncorrelated variables and a stable ranking for most classifiers. Louise et al. [103] also use the ML classifiers XGBoost, RF, SVM, DT, and LR for a four-way classification and summary plots from SHAP were used to visualise and interpret the ML models and also show biologically plausible results. Also, SHAP force plots were used to investigate individual predictions of interesting subjects. The study shows a moderate to significant correlation between the importance of natural and permutation features in SHAP value comparisons. Another study by Gaur et al. [109] uses the ML models LR, SVM, KNN, Multilayer Perceptron, and DT for a two-way classification along with LIME and SHAP explainers. The authors were able to order features in order of importance with the help of a proposed HCI model that aims to increase trust in ML models.

In this RQ, we show that LIME and SHAP are instrumental in interpreting machine learning models across different data types. Regarding numeric data, LIME proves good at local interpretability because it can change instances and fit interpretable models, showing that it is flexible across black-box models. However, its sensitivity to perturbation methods and reliance on local approximations limit its global generalization. On the other hand, SHAP gives a single measure of how important a feature is based on cooperative game theory for numeric data. This makes the data easier to understand globally and gives a full picture of how the model acts. However, challenges arise regarding computational complexity, especially for large datasets and complex models, and interpreting specific features, particularly in high-dimensional spaces. Both frameworks contribute distinct approaches tailored to numeric, text, and image data characteristics.

In all the studies for this RQ, we found LIME explainers interpreting predictions of both ML and DL models and SHAP produced a quantitative measure of features and their rankings. The RQ also helped to group the studies based on different forms of explanation for AD prediction that will be of significant use in future research.

### Limitations, challenges and future prospects

This subsection addresses the RQ4: What are the limitations, challenges, and future prospects of LIME and SHAP in AD detection?

Several studies have suggested using the concepts of LIME and SHAP to better understand the predictions made by AI systems. High-performance computers, access to the LIME and SHAP open-source frameworks, and the availability of the source code have significantly contributed to the rise of HCI systems equipped with AI. Despite the encouraging outcomes shown by independent studies, it is not surprising that these initiatives have several limitations. To stimulate further research in this area, we outline below some drawbacks and knowledge gaps in AD detection using LIME and SHAP.

1. One of the limitations of using LIME and SHAP for AD detection is the limited sample size of available data [100]. Therefore, to obtain reliable and robust explanations, a large number of data points are required, which can be challenging for medical datasets.
2. Several research articles use preprocessed and readily available datasets. AD detection demands analyzing multiple types of features, such as demographic data, cognitive test scores, and brain imaging data, which can be complex and difficult to interpret and mandates the need to be validated with a professional from the medical domain [105]. LIME and SHAP may not provide sufficient information to explain the complex interactions between these features. Therefore, to enhance the benefit to all stakeholders, it is necessary to include medical and AI experts to deduce the interpretability obtained by the XAI framework. We have not found any such studies in this review that have considered this aspect.
3. Researchers in XAI frequently rely on their intuition to ascertain a sound explanation without prior consultation with a medical expert [94]. When an intuition is detected that is inconsistent with how it is being understood, a confusion scenario results, raising doubts about how interpretability originated. The only way to avoid this is to have access to ground truth data so that one may objectively validate the explanation against it without questioning the XAI systems’ judgments.
4. Multiple XAI frameworks have been employed in some research to enhance explainability. While this may sound good from an academic point of view, it sometimes leads to ambiguity. One study combined the use of the LIME and SHAP frameworks [109]. However, there was no correlation between the feature rankings produced by these frameworks. Another study that tested the interpretability of SHAP combined it with other techniques [103]. Again, a bad association between feature ranks of the SHAP values and other models was discovered. The explanations provided by multiple XAI models may cause ambiguity, which can undermine the confidence and trust of clinicians in AI decisions as a whole, not just in the interpretations of XAI.
5. Even though researchers used XAI frameworks to predict AD, there is always a tradeoff between the interpretability of a model and accuracy. While LIME and SHAP can improve the interpretability of a model, they may reduce the accuracy, particularly of complex models [91]. Therefore, it is important to balance interpretability and accuracy when using these techniques.

Several studies have offered to explain AD prediction and subsequent interpretation using the LIME and SHAP frameworks. While the reviewed articles demonstrated significant progress in achieving clinical accuracy, the raised RQs, along with acknowledged limitations and challenges, underscore the need for more targeted research endeavors. This is pivotal for driving substantial improvements in XAI-based AD systems in real-world medical scenarios. For instance, envision a hospital setting where clinicians rely on an XAI-based AD classification model to interpret and validate a complex data sample of an individual. In this scenario, the XAI framework should provide clear and understandable explanations for why the model arrived at specific predictions, guiding clinicians in making informed decisions about patient care. This kind of contribution is yet to be carried out due to the non-availability of large datasets and XAI ground truth data. On the other hand, AI researchers must thoroughly study the issues discussed in RQs, keeping medical professionals in the loop to provide the medical community with profound reliability and trustworthiness for AI-driven AD diagnosis.

## Conclusion

A carefully selected set of research questions guided this systematic review of 23 articles that utilised LIME and SHAP for AD classification. It gave us a comprehensive understanding of these XAI frameworks’ advantages, obstacles, and prospects for AD detection and classification. Our findings not only highlight the potential of these frameworks to enhance the interpretability of AI models for AD detection and classification but also underscore the need for ongoing research and development to address the challenges and limitations of these methods. Our review will inspire further research in this critical area and help advance our understanding of how XAI frameworks can be leveraged to improve the diagnosis and treatment of AD.

## Figures and Tables

**Fig. 13:**
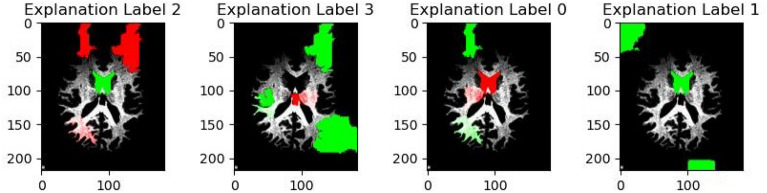
LIME-Output of code Walkthrough

**Fig. 14:**
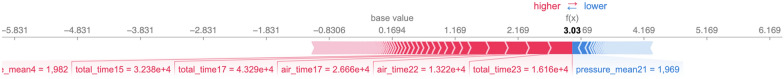
SHAP-Output of code Walkthrough (Force Plot)

**Fig. 15:**
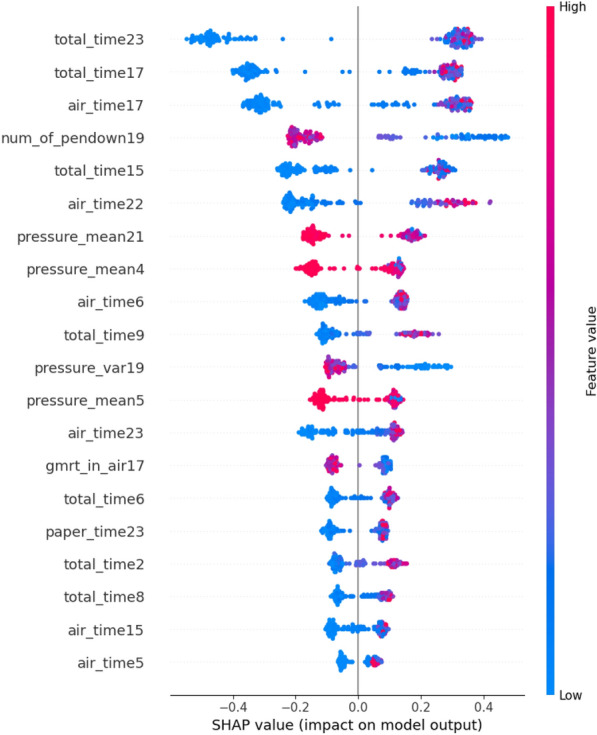
SHAP-Output of code Walkthrough (Summary Plot)

## Data Availability

The data used in code walkthrough can be obtained by contacting any of the authors.

## Appendix: Code walkthroughs

This section provides code walkthroughs for implementing the LIME and SHAP frameworks. The code shown in Listing 1 implements the LIME framework that provides local explanations for AD classification using MRI scans for a CNN model. The code in Listing-2 demonstrates implementation of the SHAP framework for enhanced interpretability for an XGBoost model that predicts AD using tabular data.

## Code walkthrough: LIME XAI framework

Lines 1–15 represent the required dependency packages. Line 16 defines a function that groups train and test data from an h5 file which is invoked in Line 17. The h5 file stores MRI image data and labels for training and testing as a numpy array. Lines 18–21 code snippet prepares data for deep learning. The x_train and x_test data are converted to a 3D format suitable for pretrained deep learning models. The y_train and y_test data containing categorical labels is converted to one-hot encoded vectors.

Lines 22–39 define a CNN model named model for image classification using the InceptionV3 pretrained architecture. The pretrained InceptionV3 model is loaded with weights from ImageNet and configured to exclude the top classification layer (Line-23). The layers of the pretrained model are frozen to retain their learned features during training (Line-24). The model is then extended with additional layers, including dropout for regularisation, batch normalisation for stable training, and dense layers for classification (Lines 25–37). The last layer has four units with softmax activation for multi-class classification (Line-37). The model summary shows the architecture and the number of parameters (Line 38). Finally, the weights of the model are loaded from a file named best-weights.hdf5 (Line-39).

The code in Lines 40–46 retrieves an MRI image from the x_train array, reduces its dimensions to eliminate singleton dimensions using np.squeeze and then displays the grayscale image using matplotlib commands. After displaying the image, it is saved as a JPEG file named ‘output.jpg’ using the plt.imsave function, with the ‘grey’ colourmap specified to ensure the grayscale format is preserved in the saved image.

The code in Lines 47–57 reads the previously saved JPEG image output.jpg using the PIL library’s Image.open function, resizes it to the required dimensions and converts it to a numpy array. The array is then preprocessed, expanding its dimensions and making it suitable for input to a model. The deep learning model, denoted as model, predicts the class probabilities for the image (Line 52), and the predicted label is obtained by finding the index with the highest probability (Line 54). The true label of the image from the y_train array is printed for comparison (Line 57).

In the code snippet from lines 60–66, a LimeImageExplainer is employed to interpret the model’s prediction for a preprocessed MRI image. The LimeImageExplainer generates an explanation for the top 4 labels, creating a visualisation with a mask overlay on the original image to highlight influential regions (see Fig. 13). The displayed image, along with the true, predicted, and explanation labels, facilitates a comprehensive understanding of the model’s decision-making process. By showcasing the areas that contributed to the prediction, this explanation aids in interpreting and validating the model’s behaviour in the context of the given MRI data.

## Code walkthrough: SHAP XAI framework

Lines 1–5 represents essential libraries for numerical operations, data manipulation, and visualisation. The subsequent steps involve preparing a dataset, initialising an XGBoost model, and integrating the SHAP library to gain insights into feature importance.

Line 6 in the code initiates the SHAP library for JavaScript components required for SHAP visualisations. Additionally, Line 7 sets a specific seed value (12345) to control the randomness in the subsequent processes, ensuring reproducible results across multiple runs.

The code in Lines 8–11 is used as an example to introduce the SHAP library and its functionalities, demonstrating the loading of a dataset and providing a visual representation of its contents. Line-8 imports data from a CSV file into a pandas dataframe. The dataset includes handwriting data from 174 participants. The classification task consists in distinguishing AD patients from healthy people. The features (X) related to handwriting analysis attributes and a target variable (y) indicating whether a person is healthy or AD. Line 9–10 prepare the data by separating features and the target variable, making it suitable for training a machine learning model.

The train_test_split function from the scikit-learn library in Line-11 facilitates the process of partitioning the dataset into two disjoint subsets. The training set (X_train, y_train) is used to train the model, while the validation set (X_valid, y_valid) serves as an independent dataset for evaluating the model’s performance. The test_size parameter specifies the proportion of the data to allocate to the validation set, and the random_state parameter ensures reproducibility by fixing the random seed for the split.

The code segment in Lines 12–16 encapsulates the initial steps of configuring an XGBoost model, preparing data, and establishing hyperparameters for training. Lines 12–13 are used for data preparation using the DMatrix objects, which are specific data structures used by XGBoost. ‘dtrain’ and ‘dvalid’ represent the training and validation data objects respectively. Line-14 provides an initial prediction score for all instances and Line-15 sets up the hyperparameters for training. Line-16 creates a ‘watchlist’ to monitor the performance of the XGBoost model while training. This is a diagnostic tool consisting of a list of tuples, where each tuple is DMatrix object with a corresponding label.

Line 17 involves the actual training of the model and evaluating its performance on the validation set. The params argument includes various hyperparameters that control the learning process as initialised in Line 15, dtrain is the training dataset provided as a DMatrix object, num_boost_round=5000 specifies the maximum number of iterations during training, evals=watchlist indicates the dataset (watchlist) on which the model’s performance will be evaluated during training, early_stopping_rounds=20 enables early stopping, where training will stop if the performance on the validation dataset does not improve after 20 consecutive rounds, and verbose_eval=100 specifies that the training progress will be printed to the console every 100 iterations. The model object will contain the trained XGBoost model after the completion of this process.

The code in Lines 18–19 sets up a TreeExplainer for the XGBoost model (created in Line-17) and then calculates Shapley values for the entire dataset to find the feature contributions and their impact on the model’s predictions. The TreeExplainer function creates a SHAP object, explainer, to explain the output of the tree-based model. The statement in Line-19 creates Shapley values for the entire dataset X that represents the contribution of each feature. Shapley values provide insights into the importance and impact of each feature on the model’s predictions. Positive Shapley values indicate a positive contribution to the prediction, while negative values suggest a negative contribution. These Shapley values can be used to interpret the model’s decision either for individual instances or to analyse the overall feature importance of the dataset.

The code in Line 20 creates a force plot of the first instance using the SHAP library to give a visual representation of the XGBoost model’s prediction (see Fig. 14) and Line-21 gives a summary plot (see Fig. 15). The explainer.expected_value is the model’s expected output, and shap_values[0,:] are the Shapley values for the first instance. The X.iloc[0,:] represents the feature values of the first instance. The visual representation shows the contribution of each feature to the model’s prediction for a specific instance, aiding in the interpretability of the XGBoost model. This line can be iterated using a loop to show the force plot of any number of instances.

In this walkthrough of Explainable AI using LIME and SHAP, we explored techniques to interpret complex machine learning models. LIME, a model-agnostic approach, provided local explanations for AD classification on MRI images. SHAP, rooted in cooperative game theory, enhanced interpretability for an XGBoost model predicting AD using handwritten data. These tools, offering both local and global insights, contribute to model transparency and trust, crucial for real-world applications like healthcare and finance. As XAI advances, LIME and SHAP play pivotal roles in making AI more understandable and accountable.
